# Nuclear Quantum Effects Made Accessible: Local Density Fitting in Multicomponent Methods

**DOI:** 10.1021/acs.jctc.3c01055

**Published:** 2023-11-03

**Authors:** Lukas Hasecke, Ricardo A. Mata

**Affiliations:** Institute of Physical Chemistry, University of Göttingen, Tammannstrasse 6, 37077 Göttingen, Germany

## Abstract

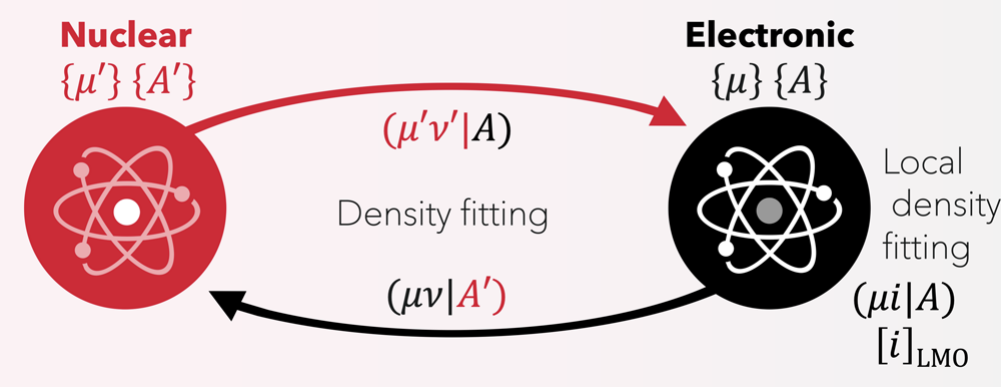

The simulation of nuclear quantum effects (NQEs) is crucial for an accurate description of systems and processes involving light nuclei, such as hydrogen atoms. Within the last years, the importance of those effects has been highlighted for a vast range of systems with tremendous implications in chemistry, biology, physics, and materials sciences. However, while electronic structure theory methods have become routine tools for quantum chemical investigations, there is still a lack of approaches to address NQEs that are computationally accessible and straightforward to use. To address this, we present the first combination of the nuclear-electronic orbital Hartree–Fock approach with both local and density fitting approximations (LDF-NEO-HF). This results in a low-order scaling approach that enables the inclusion of NQEs for large systems within a fraction of a day and for small to medium size systems in minutes. Moreover, we demonstrate the qualitative accuracy and robustness of our approach to retrieve NQEs for three real-use cases motivated by chemical, biological, and materials science applications.

## Introduction

In conventional quantum chemical calculations, the Born–Oppenheimer (BO) approximation is commonly invoked to separate the electronic and nuclear degrees of freedom. From there, the nuclei are oftentimes propagated classically on the electronic potential energy surface, or approximations are taken to extract dynamic nuclei information. However, nuclear quantum effects (NQEs) can become sizable in a variety of situations, including but not limited to conical intersections, molecular vibrations, and low-temperature atomic coherence and exchange effects.^2^ This is particularly true when dealing with light nuclei, as even at room temperature, NQEs cannot be easily disregarded. The substantial coupling between the nuclear and electronic wave functions directly influences the phenomena being investigated. Given how hydrogen atoms and proton are ubiquitously found in chemistry, this generates a need for specific methodologies for this class of nuclei alone. Several approaches have been proposed for the simulation of NQEs, some more tailored to the description of light nuclei and others less. These include path integral molecular dynamics (PIMD)^3,4^ and Gaussian wave packet dynamics,^5^ just to name a few. In hybrid approaches, some nuclei are handled quantum mechanically, while the other nuclei are propagated on vibrational or vibronic surfaces using a nonadiabatic method. Multicomponent methods use wave function or density functional theory to quantum mechanically handle nuclei, usually protons, on the same level as electrons.^6^ Inherently included are the anharmonic effects of the complete system as well as nuclear delocalization, zero-point vibrational energies, and tunneling of the quantum nuclei. The nonadiabatic interactions between electrons and quantum nuclei are included without the need for BO separation. Based on the original idea from Thomas^7,8^ and the pioneering works by the groups of Parr and Gross,^9,10^ several different branches/implementations of this multicomponent treatment have been developed. Some examples include the dynamical extended molecular orbital, the nuclear orbital molecular orbital, the multicomponent molecular orbital, and the electronic and nuclear molecular orbital approaches.^11−16^

In this work, we will make use of the denomination nuclear-electronic orbital (NEO) approach, as used by Hammes-Schiffer and co-workers. Over the years, the NEO ansatz has been applied not only to density functional theory (NEO-DFT)^17−26^ but also to a myriad of wave function methodologies.^27−31^

Although such methods hold great promise for the routine treatment of NQEs, their application has been somewhat limited. In part, this is due to their increased computational cost. Implementations available today present sizable overheads, and the computational scaling of NEO wave function methods is extremely unfavorable.^29,30^ Adding to this, some NEO implementations have shown convergence issues due to the interplay between the electronic and nuclear self-consistent field (SCF) cycles.^32^ Recently, a second-order SCF algorithm was developed by Reiher and co-workers to enhance the stability and computational costs of the convergence procedure.^32^ Additionally, Hammes-Schiffer and co-workers developed a simultaneous optimization algorithm for the wave function for both the electrons and quantum nuclei by exploiting the direct inversion of iterative subspace, leading to substantially reduced computational costs. This works well for cases with a dominating nuclear quantum system.^33^ However, these are issues that arise only when all quantum nuclei are treated with a single Fock matrix. Under the distinguishable (or independent) particle approach, exchange energy between nuclei is neglected, as these values are commonly even below the energy convergence thresholds.^26^ Each quantum nucleus is treated with an independent set of equations, meaning a Fock matrix for each nucleus. This is generally recommended for all multicomponent implementations, but if for some reason (algorithmic dependencies, description of nuclear collisions, etc.) the independent particle approximation cannot be used, then one should be careful with the construction of the starting nuclear density guess. Within this work, we present a block-diagonalization approach for an improved starting guess. An alternative implemented in other algorithms is the use of atomic like nuclear starting guesses.^33^

Another possibility to make NEO calculations more amenable is the use of Cholesky decomposition. The latter has been applied in the context of NEO-DFT,^34^ but dramatic savings in computational time have yet to be demonstrated. Density fitting approximations have also been recently introduced for NEO-DFT^29,30,35^ but with only moderate computational savings. Density fitting has been used for correlated methods (NEO-MP2, NEO-SOS′-OOMP2, and NEO-CCSD),^29,30^ with more visible gains, as expected due to the large number of four-index integrals. Still, the usefulness of density fitting for NEO reference calculations has yet to be clearly established.^6^

**Figure 1:**
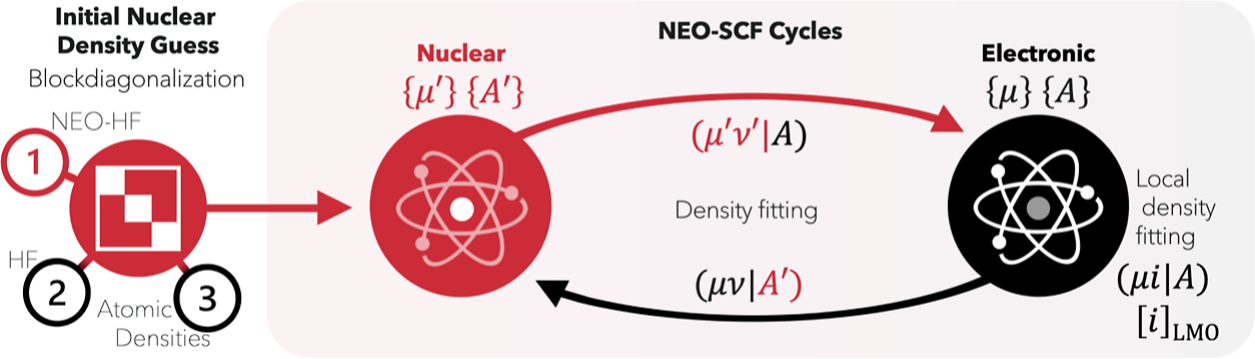
Overview of the LDF-NEO-RHF algorithm. The initial quantum nuclei density guess is provided by a block-diagonalization using an effective electronic potential. The latter can be obtained from three different sources (a previous NEO-HF calculation, a regular HF calculation, or atomic densities). For most cases studied, the atomic density guess is enough to provide a stable SCF convergence pattern. The coupling between the quantum nuclei and the electronic subsystems makes use of the fitting basis sets to accelerate the calculation of the Coulomb integrals. The electronic calculation makes use of local density fitting.^1^ Four basis sets are required: two atomic ({μ}, {μ′}) and two auxiliary ({*A*}, {*A*′}) fitting bases.

In this contribution, we present and discuss the implementation of local density fitting approximations for use in NEO calculations, circumventing the main computational bottlenecks, effectively making these approaches as accessible as conventional electronic structure theory. The algorithms discussed are of interest for both NEO-DFT and wave function calculations, although we will focus on NEO-Hartree-Fock (NEO-HF) calculations, the basis for every type of wave function-based NEO approach. An overview of the main components are shown diagrammatically in Figure 1. We show that it is possible even for medium-sized applications to reduce the computational cost by more than 1 order of magnitude with negligible deviations to canonical NEO results. We show the usefulness of the algorithm for selected applications, effectively demonstrating that this advance places multicomponent calculations into the standard quantum chemical toolbox, much beyond what is observed in the literature so far. The fundamentals of our approach and particular algorithmic details are presented in the following. All of the points discussed can be straightforwardly implemented in standard quantum chemical packages. Our implementation is provided in the Molpro package.^36^

## Methods

### NEO Hartree–Fock Theory

In order to account for NQEs in a computationally efficient manner, the NEO approach is employed in this work.^6^ In the following, the NEO-restricted Hartree–Fock (NEO-RHF) theory is briefly outlined.^27^ In NEO-RHF, the complete system is divided into two subsystems. The first system contains all the particles, *N*_e_ electrons and *N*_n_ nuclei, which are treated quantum mechanically, while the other system consists of the classical nuclei *N*_c_. Using the separation between the quantum mechanical treated electrons and nuclei and the BO approximation, the regular electronic Hamiltonian operator is expanded to accommodate the quantum nuclei
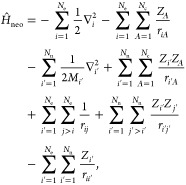
1in which primed indices are indicating quantum nuclei and regular indices refer to electrons. Additionally, the reference wave function for the multicomponent system
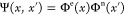
2is built as a product of individual Slater-type wave functions for the Fermionic nuclear system Φ^n^(*x*′) and electronic system Φ^e^(*x*), with the respective coordinates. Utilizing the representation in basis functions, this leads to a coupled pair of Roothaan–Hall equations
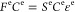
3
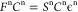
4where the Fock operators are given, in the formulation of the regular one-particle *H* and two-particle *G* matrix elements, as
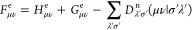
5
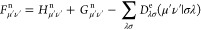
6

Therefore, the construction of the Fock matrix for both subsystems requires the density matrix
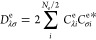
7
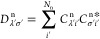
8build from the respective molecular orbital (MO) coefficients *C* of the coexisting quantum mechanical subsystem, as indicated by the last Coulomb coupling terms. The distinguishable (independent) particle approximation can be applied by constructing individual Fock matrices for each quantum nucleus, limited to the basis space of each atomic center.

### Conventional Density Fitting NEO-RHF

We start by discussing conventional density fitting in NEO-RHF. Albeit this has been implemented in other multicomponent algorithms,^35^ we observe the most significant speed-ups reported to date. Therefore, we find it relevant to discuss our specific implementation in addition to the local approximations which are later introduced.

In order to alleviate the computationally demanding Fock matrix construction, necessary in both NEO-HF and NEO-DFT with hybrid functionals, density fitting can be employed. Through the use of density fitting, the computational effort scales asymptotically with  for the Coulomb contributions and  for the exchange contributions, with respect to the molecular size *N*.^37^ In this approximation, the four-center two-particle integrals
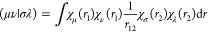
9consisting of atomic basis functions {χ_μ_} are computed in terms of 2-index Coulomb metric
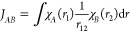
10and 3-index integrals
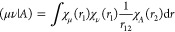
11where {χ_*A*_} are denoting auxiliary fitting basis functions. In order to approximate the one-particle density, expressed by an orbital product density ρ_μν_(*r*) = χ_μ_(*r*)χ_ν_(*r*), an expansion in the fitting basis functions
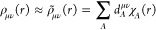
12is utilized. There are different ways to determine the expansion coefficients, but in our case, this is done by minimizing the least-squares error of the electric field.^38^ This is presented in further detail in the following section together with the working equations. Specific to NEO, the coupling Coulomb terms are density-fitted as well and then inserted into the respective electron or nuclear Fock matrices, depending on the SCF cycle being carried out.

### Robust Density Fitting

In our implementation, the expansion coefficients for the auxiliary densities in eq 12 are determined by the minimization of the positive definite functional
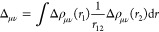
13where Δρ_μν_(*r*) = ρ_μν_(*r*) – ρ̃_μν_(*r*) is the difference between the exact and approximated one-particle density.^39^ By using the weight operator , the least-squares error of the electric field is accurately minimized.^38^ As a result, the expansion coefficients are obtained as
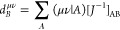
14which leads to the following expression for the four-center two-particle integrals
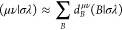
15
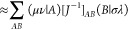
16

As a result, the Coulomb part of the density-fitted Fock matrix is now computed as
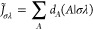
17with the vector *d*_*A*_
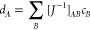
18where
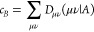
19is the contraction of the 3-index integrals with the density matrix. In addition, exchange contributions to the density-fitted Fock matrix can be computed as
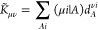
20where the expansion coefficients are given as
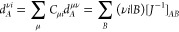
21and
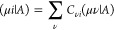
22are half-transformed 3-index integrals.

### Local Density Fitting LDF-NEO–RHF

With the aim of reducing the asymptotic scaling of the exchange contributions even further, local density fitting can be employed. Therefore, the fast and robust localization to intrinsic bond orbitals is employed.^40^ As a result, the sparsity of the electronic MO coefficients can be exploited in the localized molecular orbital (LMO) basis, and the exchange terms are given as the sum of the contributions from individual MOs. In this localized basis, the half-transformed 3-index integrals transform with the LMO coefficients to
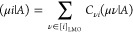
23where μ ∈ [*i*]_AO_ and *A* ∈ [*i*]_fit_. In this notation, [*i*] refers to the orbital specific subset of basis functions also called domains. As a result of the division in distinct domains, an asymptotic scaling can be achieved for the summation over all LMOs since the individual domains are independent of the molecular size *N*. However, this only holds true for large molecules where the LMO domains tend toward a constant size and the conjunct AO domains become well separated.^41^ For small- to medium-sized molecules, the AO domains are far-reaching, and therefore, the computational savings are in the worst case reduced to a  scaling with a low prefactor.^1^

### Determination of NQEs from (L)DF-NEO-RHF

In order to capture NQEs with the computational efficient (L)DF-NEO-RHF method but also including electron–electron correlation, we apply a composite approach. We correct higher level electronic single-point energies with one additional multicomponent calculation, as
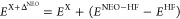
24where *E*^X^ is the energy of a DFT/correlated wave function method, and the correction for NQEs is obtained as the energetic difference of a multicomponent *E*^NEO–HF^ and regular *E*^HF^ Hartree–Fock computation.

### Implementation Details

With the intention of developing a fast, accurate, and generally applicable (L)DF-NEO-RHF implementation, which enables the incorporation of NQEs for large systems in post-HF treatments, we build our program with the efficient density-fitting Molpro routines.^1,37,42^ For most applications, the major computational bottleneck remains the electronic structure problem. In order to ensure a maximum speed-up of the electronic SCF computation for mid- and large-sized systems, we implemented density fitted and, respectively, local density-fitted approximations in our NEO-RHF program. The SCF cycles of both quantum mechanical subsystems can be carried out stepwise or simultaneously, whereas both schemes approach the same computational costs in the case of a dominating electronic system with indistinguishable quantum nuclei.^33^ We opted for a stepwise SCF implementation, which furthermore allows a complete basis set separation between the electronic and nuclear basis sets within the Molpro framework and thus speeds up the computation further.

The size of the electronic structure problem, however, will also reflect on the coupling contributions, which becomes the second most expensive computational step for larger molecules. The latter contributions must be computed once after the SCF procedure of the respective subsystem is converged. Since the coupling expression only includes the Coulomb contribution of the electronic and nuclear attraction (eqs 5 and 6), density fitting reduces the computational costs to an asymptotically quadratic scaling. The least computationally demanding part will be the nuclear quantum system since the relevant quantum nuclei represent only a small fraction with respect to the overall number of particles in the system. Therefore, an integral direct implementation with density matrix element prescreening is the default for the nuclear SCF iterations.^42^ With respect to the relative localization of the nuclear density, also recently shown by Reiher and co-workers, a maximum speed-up can be achieved through the prescreening process.^32^ Following the first proposed NEO-RHF implementation, the algorithm starts with the nuclear SCF which requires an initial electronic density to form the coupling contribution of the Fock operator (in eq 6).^27^ While those initial electronic densities are commonly obtained through prior conventional RHF computations, it is also possible to construct the electronic starting density as natural orbitals from a diagonalized electronic density matrix. The latter is obtained from the atomic orbitals and atomic occupation numbers. This approach avoids the additional costs of a prior RHF calculation completely, which is especially beneficial for large systems. However, for complex electronic structures, this can be offset by a slower convergence behavior. An alternative is to start from a minimal basis set and project the solution to the target full basis, which ensures robust and fast convergence.

**Figure 2:**
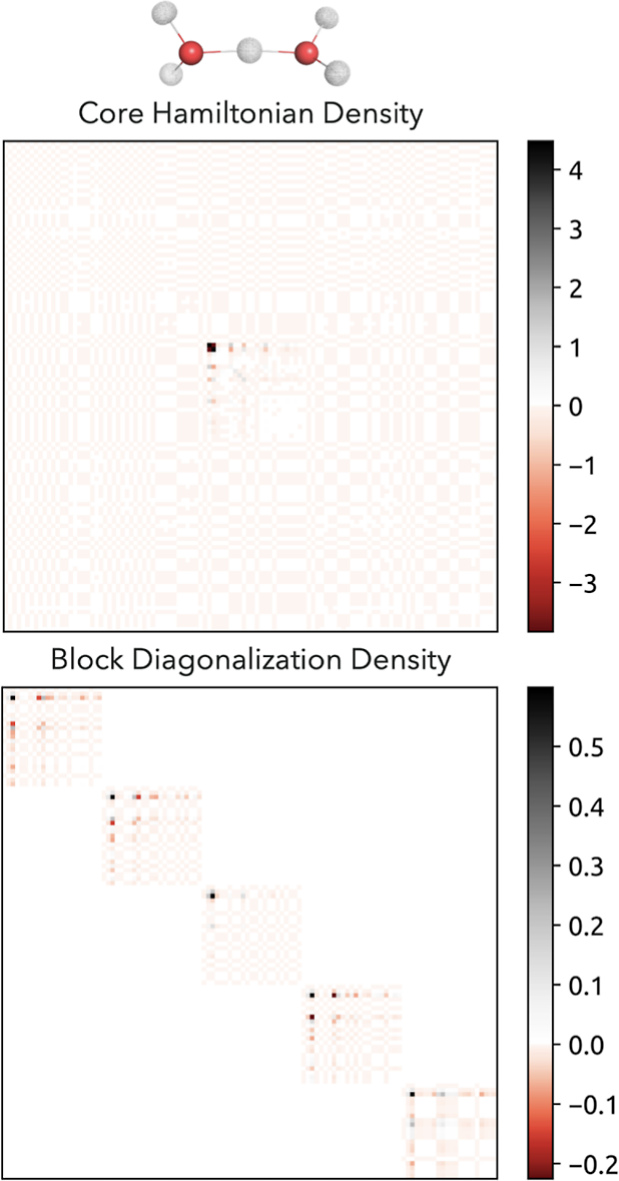
Example of the initial nuclear densities for H_5_O_2_^+^ obtained through a full diagonalization of the nuclear core Hamiltonian (upper part) vs a block diagonalization (lower part), where the corresponding color map illustrates the density matrix elements.

We would like to comment further on the construction of the starting guess, as this is scarcely mentioned in the literature. The construction of the initial nuclear density, if not projected from a prior NEO-RHF computation, is regularly computed from the nuclear core Hamiltonian. If one is not applying the distinguishable particle approximation, this can lead to serious issues. It could be, in fact, the reason behind many NEO convergence problems previously reported.^32,33^ Without this approximation, several nuclei may potentially localize on a single center. To avoid this, one can employ a block diagonalization over each nuclear center even if the independent particle approximation is not used. The two alternative initial density matrices (obtained via full vs block diagonalization) are illustrated in Figure 2. In the case of a full diagonalization, the protons are mostly localized at one nuclear center since there is no repulsion between quantum nuclear densities in the core Hamiltonian. In the case of the block diagonalization, one starts rather close to the converged solution, where every nuclear center is occupied.

## Computational Details

In all SCF computations, we employ the direct inversion in the iterative subspace as a convergence accelerator starting after the first iteration with 10 Fock matrices as a basis to extrapolate.^43,44^ As convergence criteria, we employ the defaults integrated in Molpro, namely, the energy difference, the density difference, and the gradient. For the water clusters, a threshold of 10^–8^ au is used for the energy difference within the SCF computations, the difference in the density between iterations and the gradient. All the three criteria must be fulfilled for convergence. For the minimal required energy difference in the NEO-RHF iterations, we set a threshold of 10^–7^ Hartree. For the other systems, a threshold of 10^–7^ au is used for the three convergence criteria of the NEO subiterations and 10^–6^ Hartree for the difference between the NEO-RHF iterations. As electronic basis, we employ the cc-pVTZ basis set, and for the nuclear basis, we employ the especially tailored PB4-F2 basis set for multicomponent calculations.^45,46^ For the electronic density fitting, the cc-pVXZ-JKFIT basis set at the X = T triple-ζ and X = Q quadruple-ζ level is employed.^47^ The density-fitted coupling contribution in the nuclear iteration is computed with the 10s10p10d10f even tempered basis set with exponents ranging from  to 64. The PNO-LCCSD(T) calculations were carried out with Molpro 2023.1 using the cc-pVTZ basis set and the default settings.^36,48,49^ For the optimization of the two molecular chains and the water tetramers, the ORCA 5.0.3 program was used with the B3LYP/G functional and the def2-TZVP basis set.^50,51^ The optimization settings were adjusted with the TIGHTOPT keyword and corrected for dispersion via the D3 method with Becke–Johnson damping.^52,53^ Moreover, the *C*_2*h*_ and *D*_2*h*_ symmetries of the two chains were exploited. Additionally, the chains with 6–9 quinonediimine molecules used for the performance benchmark are assembled according to the canted chain scheme of Cahlík et al.^60^ and optimized with HF-3c.^54^ The DNA performance benchmark models were built on the structures obtained from the DNA sequence to the structure generator of the Supercomputing Facility for Bioinformatics & Computational Biology IT Delhi based on conformational parameters taken from experimental fiber diffraction studies.^55^ The structures were protonated, and the protons were subsequently optimized with HF-3c. The volume visualization of the nuclear density was performed with the ParaView 5.11.1 program, whereas the couture representation of the nuclear density was performed with the PyMOL 2.5.2 program.^56,57^ All benchmark calculations were computed on a commercially available IntelXeonGold 6342 2.80 GHz CPU with 48 threads.

## Results

### DF-NEO-RHF Performance

In order to benchmark the accuracy and speed-up of the DF-NEO-RHF method, we employ as first test systems a series of size-increasing protonated water clusters. These clusters exhibit sizable NQEs and are well established for benchmarking the performance and advantages of multicomponent methods.^32,58^ We start by comparing the errors in the absolute energies from DF-NEO-RHF relative to those of a complete integral-direct NEO-RHF implementation. Prior, the integral-direct version was benchmarked against the Q-Chem 6.0 NEO-RHF implementation, where all energies agree within the set threshold of 10^–8^ Hartree.^59^

**Figure 3:**
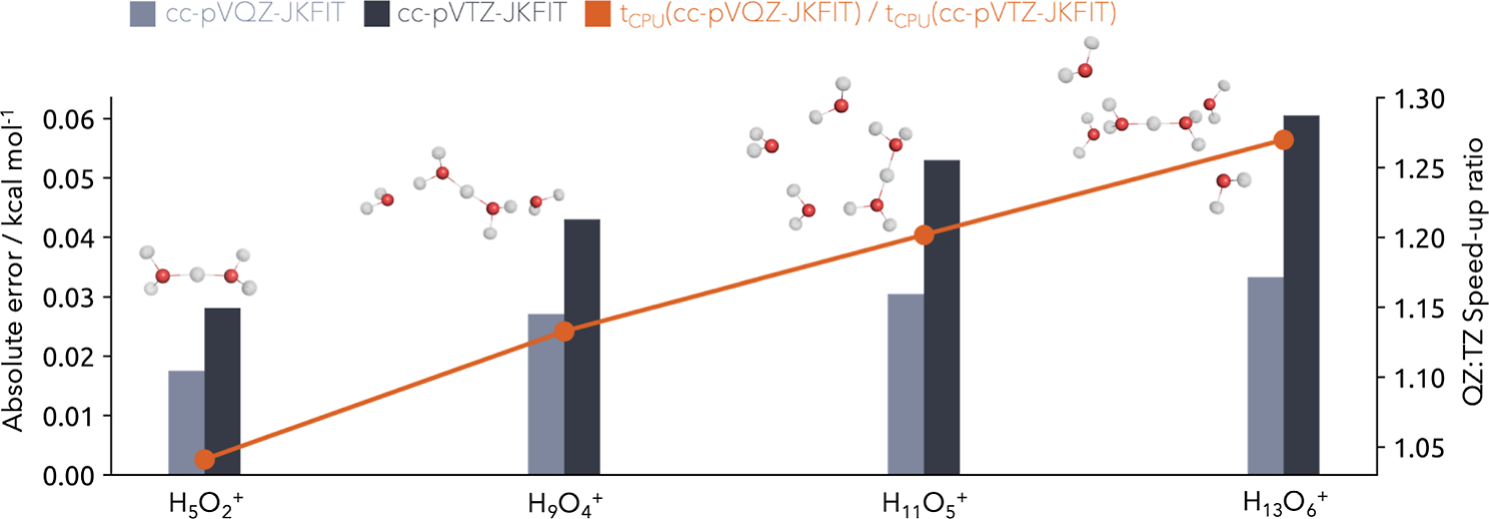
Absolute error of the density fitting (light and dark gray) in comparison to integral-direct reference computations as well as the corresponding computational speed-up (orange) for the cc-pVTZ-JKFIT and cc-pVQZ-JKFIT fitting basis sets for multiple size-increasing protonated water clusters. All protons of the clusters are treated quantum mechanically, and the nuclear density is shown at a 0.01 σ contour level.

Two sets of density fitting basis sets are used in this comparison. In addition to the error analysis, the corresponding computational speed-up ratio in relation to the fitting basis sets size is shown in Figure 3. For the extended fitting basis set, the overall density fitting error is as expected smaller, 0.018–0.033 kcal mol^–1^ compared to 0.028–0.060 kcal mol^–1^ with the smaller fitting bases. Overall, with respect to the system size, the errors of both fitting basis sets increase consistently slow and are in agreement to preceding analysis of density fitting errors for correlation methods within the NEO framework carried out by the group of Hammes-Schiffer.^30^ By choosing the smaller fitting basis set with the trade-off of slightly lower accuracy, it is possible to achieve additional speed-ups ranging from 4 to 27% for the smallest to largest water cluster. The effect will, of course, be larger for the extended systems.

As previously mentioned, concerns have been raised about convergence issues in NEO-SCF calculations.^32^ These occur when the distinguishable particle approximation^26^ is not in use. Nonetheless, these can be avoided by block diagonalization of the core nuclear Hamiltonian. Thereby, the localization of the nuclear centers which leads to a block structure of the nuclear density, also shown by Reiher and co-workers is exploited.^32^ This approach leads to a substantial reduction in the number of iterations. In the case of water clusters, the total number of nuclear iterations in the first NEO cycle can be reduced by 5–14 times. For larger systems, we found it even impossible to reach convergence if the block structure was not exploited. In summary, this corresponds to a distinguishable particle approximation for the starting guess.

**Figure 4:**
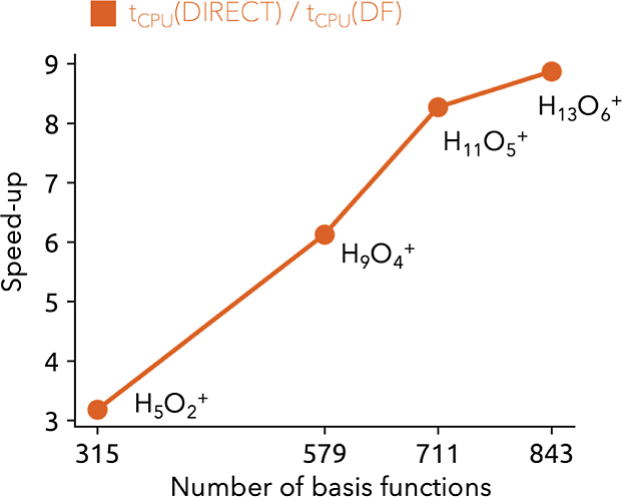
Computational speed-up of the density fitting approach in comparison to the integral direct version for multiple size-increasing protonated water clusters.

Although the reduction of nuclear iterations is tremendous, the resulting speed-up for large systems is strongly influenced by the density fitting approximations in the electronic SCF. The overall speed-ups of the density fitting approximations in the electronic and coupling part of the NEO-RHF method against the integral direct version for the water clusters are shown in Figure 4. Even though the absolute system sizes of the water clusters are considered comparatively small for drastic speed-ups from density fitting approximations in the electronic part, density fitting for the Coulomb coupling enables even for smaller systems an impressive overall speed-up, a factor between 3 and 9, for the trial water clusters compared to the integral direct version. The absolute DF-NEO-RHF computational time ranges from 1.5 min for the smallest water cluster to 4 min for the largest water cluster.

### Protonated Water Tetramers

**Figure 5:**
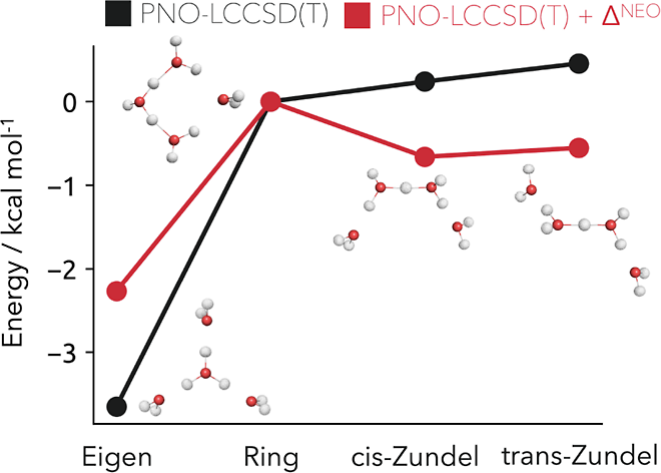
Relative stability of the four distinct protonated water tetramers computed with local CCSD(T) (black) and corrected by the NQEs from an NEO calculation (red). All nine protons of the isomers are treated quantum mechanically, and the nuclear density is shown at a 0.01 σ contour level.

In the following, we will briefly illustrate a powerful advantage of this low-cost DF-NEO-RHF method on the example of protonated water tetramers. For these tetramer structures, the commonly used gold standard for electronic structure calculations in a theoretical chemistry-coupled cluster with single and double excitations and perturbative triples CCSD(T) fails to predict the qualitative energetic ordering of the four isomers as shown in Figure 5.^30^

However, by correcting the PNO-LCCSD(T) reference energies by the DF-NEO-HF result (eq 24), the relative qualitative correct ordering is achieved despite minor energetic differences in the cis- and trans-Zundel ordering. In general, this demonstrates the superiority of this method as a first corrective approach to high-level computations to capture the impact of NQEs for a plethora of systems. In order to systematically improve the obtained results, correlation energies of proton–electron interactions can be included toward a true quantitative description, but this is left for future work. With the aim to provide those correlation corrections also for large systems, an efficient local implementation is currently developed and benchmarked in our group. The DF-NEO-RHF method already provides an elegant approach to access NQEs in small systems at extremely low computational costs. However, for small systems, the major fraction of this speed-up arises from the asymptotically quadratic scaling of the density fitted Coulomb coupling. With respect to midsize systems, the major speed-up will origin in the density fitting of the electronic part, which can further be accelerated through local approximations in order to make large systems accessible by the NEO approach as demonstrated in the following.

### LDF-NEO-RHF Performance

With the aim to demonstrate the efficiency of the LDF-NEO-RHF method, we depict two polymeric model systems with sizable NQEs. On the one hand, these offer the advantage to be easily size scalable, which is important for benchmarking the performance of the implementation. On the other hand, the depicted systems are already thoroughly investigated concerning their dependence on NQEs. Our first test system is a two-dimensional network of quinonediimine molecules, where NQEs visibly impact the structure and are relevant for the development of new engineered materials in the future.^60^ The second example is a DNA fragment build from guanine–cytosine base pairs, unarguably one of the most important molecules from biochemistry.^61,62^ It should be noted that in the following calculations, we only compare local (LDF) to conventional (DF) implementations, as integral direct calculations would be prohibitively expensive.

**Figure 6:**
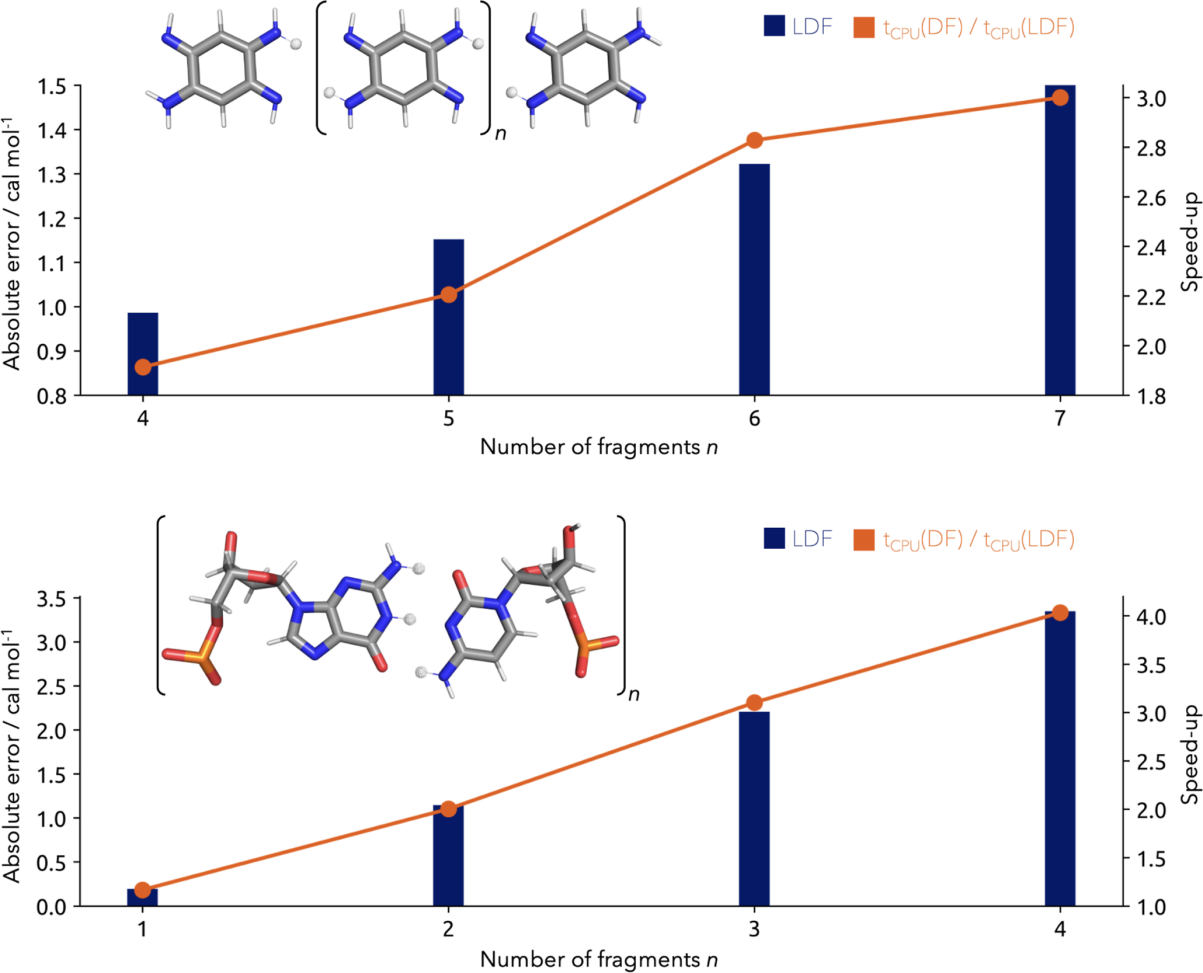
Absolute error of the localization procedure in comparison to the regular density fitting results (blue) and resulting computational speed-up (orange) for the size increasing systems of the quinonediimine chain (upper part) and the DNA strand of guanine–cytosine base pairs (lower part). For the quinonediimine chain, 4 up to 16 protons and for the DNA strand 3 up to 12 protons are treated quantum mechanically, and the nuclear density is shown at a 0.01 σ contour level.

The benchmark results for both systems are shown in Figure 6. In the case of the two-dimensional quinonediimine system, we investigated chain lengths reaching from 6 up to 9 molecules. The overall error of the localization procedure reaches from 0.986 to 1.501 cal mol^–1^. Considering the error of the density fitting procedure itself, the local approximation can be considered as extremely accurate. In addition to the precise localization, the gained speed-up compared to the conventional density fitting implementation reaches from 1.9 for the shortest chain with six molecules up to 3.0 times for the longest chain with nine molecules. It should be noted that both the localization error and the speed-up scale with the system size. Notably, the speed increase increases faster than the localization error. Therefore, the benefits for large molecular systems are clear. The absolute CPU times for the LDF-NEO-RHF calculations of the quinonediimines chain reached from 0.7 h for the shortest chain to 2.3 h for the longest chain. With the aim to benchmark the local density fitting in a more complex 3D system, a DNA fragment based on guanine–cytosine base pairs is employed. Overall, the strand size varies from one up to four base pairs in total. The localization error increases from 0.195 cal mol^–1^ for the single pair to 3.348 cal mol^–1^ for four pairs. In accordance with the prior results for the quinonediimine chains, the localization error is again negligibly small and increases only slowly with the system size. Hence, the localization procedure introduces at most minor inaccuracies that are orders of magnitude lower than the density fitting error for both 2D and 3D systems. After analyzing the accuracy of the local approximation, the benefit remains to be quantified. For the DNA fragments, the speed-up reaches from 1.2 times for the single base pair to 4.0 times for the largest strand consisting of four base pairs. The corresponding CPU times of these calculations rise from 0.3 h for the single base pair to 9 h for the largest strand of four base pairs. Overall, LDF-NEO-RHF computations allow us to capture NQEs for large systems even with multiple quantum protons, reaching from 10 to 16 quantum mechanically treated protons in our quinonediimine chains and 3–12 quantum mechanically treated protons in the DNA base pairs, within a tiny fraction of a day. In the following sections, we exemplify how LDF-NEO-RHF can be effectively used to replace methods which are several orders of magnitude more computationally demanding.

### NQEs in Quinonediimine Chains

**Figure 7:**
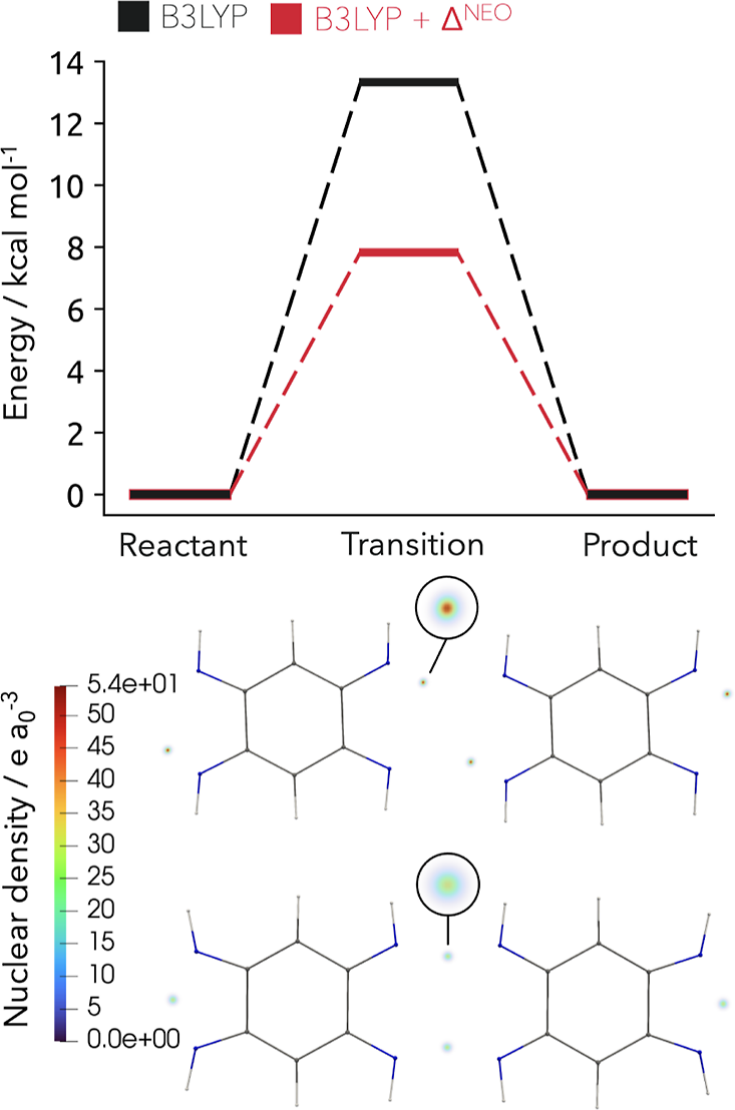
Upper part: Relative stability of the canted and straight chain corresponding to localized and transferred protons computed with B3LYP (black) and corrected by the NQEs from a NEO calculation (red). Energies are provided per proton in kcal mol^–1^. Lower part: Volume representation of the nuclear densities for the four quantum mechanically treated protons of the canted and straight chain.

Experimental and computational studies of the quinonediimine chain were carried out by Cahlík et al.^60^ A combination of scanning probe microscopy experiments and PIMD elucidated the complex interplay of NQEs in hydrogen bonds on the chemical and physical properties of the chain. While in a canted geometry of the chain, the protons are localized; they identified a second form of a straight chain in which the protons are delocalized. The impact of NQEs on the transition can be estimated as the difference between the classical MD and PIMD results. According to their simulations at 10 K, the energetic barrier for a proton-transfer event approximately reduces by half due to NQEs, and the barrier increases with higher temperatures. Here, we would like to observe if one can qualitatively describe these effects with NEO, avoiding costly molecular dynamics simulations.^63^ In order to save resources and allow for a quick and efficient prescreening of NQEs, we further tested our corrective LDF-NEO approach, which we also employed for the water tetramers, on both chain geometries. Therefore, we performed an optimization of the ground (canted chain) and transition state (straight chain), making use of the efficient B3LYP functional and performed a NEO calculation on top.^64^ The absolute CPU time for both NEO calculations was only 4.7 min. Afterward, the information from the NEO energies can be used to correct the purely electronic energies obtained by DFT. As shown in Figure 7, the incorporation of NQEs leads approximately to a halving of the energetic barrier, in line with the PIMD results. Overall, the relative lowering of the proton-transfer barrier through NQEs is with both methods qualitatively similar. In addition to the energies, the NEO method also makes the nuclear densities readily accessible. As expected, the nuclear density is sharply localized in the canted chain case with regular hydrogen bonds, whereas the straight chain shows broadly delocalized protons in shared hydrogen bonds through the proton transition. In order to provide a more accurate description of the nuclear tunneling, multistate approaches like the NEO multistate density functional theory could be used to recover the bilobal characteristic of the nuclear density at the proton transition.^65^ However, all of the latter results are obtained as a small add-on to electronic structure calculations.

### Double-Proton Transfer in DNA Base Pairs

**Figure 8:**
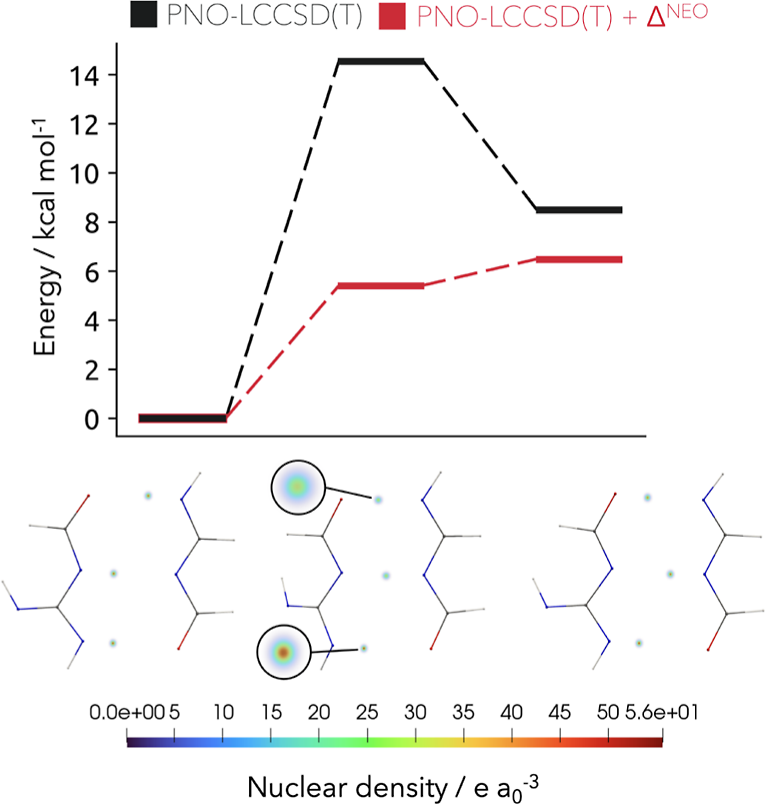
Upper part: Relative energies of the double-proton transfer for the enol tautomerization of the guanine–cytosine base pair computed with PNO-LCCSD(T) (black) and corrected by the NQEs from a NEO calculation (red). Lower part: Volume representation of the nuclear densities for the three quantum mechanically treated protons of each state during the double-proton transfer.

Mismatches in the interstrand pairing of DNA nucleobases appear to be facilitated by NQEs, namely, through intermolecular enol tautomers of guanine–cytosine base pairs.^66^ Until today, this is a highly discussed topic and for a thorough theoretical analysis and incorporation of NQEs is vital, though computationally expensive.^67,68^ One of the first studies which investigated the impact of NQEs on the enol tautomerization was carried out by Pérez et al.^61^ In order to capture the latter effects and their impact on the enol formation, they made use of the computationally expensive combination of umbrella sampling and PIMD to carry out simulations of the double-proton transfer at 300 K. As a result, they discovered that NQEs not only reduce the effective reaction barrier of the proton-transfer but also lead to a metastability of the enol tautomer. For a comparison, we used their model systems for the double-proton transfer in the guanine–cytosine base pair and computed the corresponding local coupled cluster and NEO energies. We again made use of eq 24, and the results are shown in Figure 8. The overall CPU time of the NEO calculations for all the three stages of the double-proton transfer took 7.7 min. The impact of the obtained NQEs on the stability of proton transfer is obvious. Whereas the coupled cluster results indicate a stable local minimum of the enol tautomer, incorporation of NQEs changes the overall picture. This conclusion is consistent with the prior results obtained through PIMD simulations for this system. Regarding the nuclear densities, a stabilization of the transition state via nuclear delocalization during the double-proton transfer can be found.

## Conclusions

In this work, we presented an implementation of the NEO Hartree–Fock method with unprecedented computational efficiency. Within this low-order scaling framework, it is now possible to calculate NQEs with little computational cost even for extended systems. We depicted prominent examples from vast scientific backgrounds ranging from molecular chemistry over materials science to biochemistry in order to benchmark the performance of our implementation, quality, and robustness of the obtained NQEs. As a result, the correction based on the nuclear reference energies reproduces in all tested systems the same qualitative trend as higher level calculations at a tiny fraction of the computational cost. Our implementation offers a powerful tool to elucidate the impact of NQEs on a plethora of different systems with wide-ranging applications, from materials design to drug discovery. The program is fully available within the Molpro package^36^ and will be systematically improved by multicomponent correlation methods aiming toward chemical accuracy. Currently, we are developing a composite local wave function-based approach for the NEO correlation energies with the aim to maximize the accuracy at the lowest computational cost possible. These program extensions will provide an excellent framework for the further quantitative investigation of NQEs. However, the methods presented in this work already provide a compelling instrument for more routine calculations addressing the quantum characteristic of protons.
